# Validation of the Dutch Version of the Boston Carpal Tunnel Questionnaire

**DOI:** 10.3389/fneur.2019.01154

**Published:** 2019-11-07

**Authors:** Floriaan G. C. M. De Kleermaeker, Mark Levels, Wim I. M. Verhagen, Jan Meulstee

**Affiliations:** ^1^Department of Neurology, Viecuri Medical Center, Venlo, Netherlands; ^2^Department of Neurology, Canisius Wilhelmina Hospital, Nijmegen, Netherlands; ^3^Research Centre for Education and the Labor Market, Maastricht University, Maastricht, Netherlands; ^4^Sociology Group, Nuffield College, Oxford, United Kingdom

**Keywords:** boston carpal tunnel questionnaire, carpal tunnel syndrome, validity, reliability, responsiveness, acceptability, Dutch

## Abstract

The Boston Carpal Tunnel Questionnaire (BCTQ) is a scale that has been developed specifically for carpal tunnel syndrome (CTS). It consists of the Functional Status Scale (FSS) and the Symptom Severity Scale (SSS). It is the most widely used patient reported outcome measure in CTS and has been validated in many languages. Although already widely used, psychometric properties of the Dutch version of the BCTQ are yet unknown. The aim of this study was to assess the validity, reliability, responsiveness, and acceptability of the Dutch version. Moreover, this paper focuses the longitudinal validity (the use after an intervention) of the BCTQ, which has not been investigated before. A total of 180 patients completed the BCTQ in addition to a six-point Likert scale for perceived improvement, before and about 6–8 months after carpal tunnel release (CTR). Principal factor analysis revealed that the FSS is unidimensional, consisting of a single latent factor (“functionality”) and has a high internal consistency (Cronbach's α = 0.825). However, the SSS has three dimensions, which are all highly internally consistent: “daytime symptoms” (Cronbach's α = 0.805), “nighttime symptoms” (Cronbach's α = 0.835), and “operational capacity” (Cronbach's α = 0.723). Post-treatment, the FSS still consisted of one factor, but the SSS changed in dimensionality, as it had only two factors left post-treatment. The ΔFSS and ΔSSS had good correlation with the six-point Likert scale for perceived improvement (*r* = 0.524; *p* < 0.01 and *r* = 0.574; *p* < 0.01, respectively), a moderate correlation between FSS and pinch grip (*r* = 0.259; *p* < 0.01) was found, and a weak correlation between SSS and pinch grip (*r* = 0.231; *p* < 0.01) was found. Standard Response Mean for FSS and SSS was 0.76 and 1.49, respectively. Effect size was 0.92 and 1.96, respectively, both indicating a good responsiveness. Response rate was high (82–84%). We concluded that the Dutch version of the BCTQ has a proper reliability, validity, responsiveness, and acceptability to assess the symptom severity and functional disabilities of CTS patients. Because of multidimensionality, we would recommend to create sum scores of the four different dimensions instead of two. Caution is required when interpreting the results postoperatively, due to the insufficient longitudinal validity of the SSS.

## Introduction

Carpal tunnel syndrome (CTS) is the most common entrapment neuropathy and can cause paresthesia, numbness, pain, and weakness in the territory innervated by the median nerve (1, 2). Carpal tunnel release (CTR) is considered the most effective treatment, as a favorable outcome on short term as well as long term has been demonstrated (3–5). In order to assess outcome after treatment in clinical trials, validated outcome scales are necessary. The quality of the applied scale will have a profound effect on the acquired results. It is advantageous to recall what exactly scaling instruments aim to do. Scales serve to measure traits that are an inherently unobservable concept by (1) assuming that patients' true scores on the latent concept can be quantified on a continuum, (2) presenting patients with a set of questions (items) that are positioned on that continuum, and (3) aiming to infer patients' position on that latent continuum by quantifying their answers on the items.

The Boston Carpal Tunnel Questionnaire (BCTQ) is a scale that has been developed specifically for CTS (6). It is the most widely used patient-reported outcome measure (PROM) in CTS. It is known from previous research that in CTS, standardized questionnaires like BCTQ are more sensitive to the clinical change produced by CTR than clinical examination as well as electrodiagnostic tests (7–9). Therefore, a proper version of the BCTQ is essential in clinical trials. The reproducibility, internal consistency, validity, and responsiveness of the English version have been demonstrated in the original publication (6), which has been reproduced by many other authors (10). Moreover, the BCTQ has been validated in many other languages, such as Spanish, Portuguese, Greek, Turkish, Swedish, Chinese, Korean, and Japanese (11–20). The psychometric properties of the Dutch version, however, have never been established in a CTS population, even though it has been applied frequently. Due to subtle differences in language use, the Dutch translation may have a different validity than the original version, which can lead to bias in research.

Goal of this research was to evaluate the validity and reliability of the Dutch version of the BCTQ in CTS patients. Moreover, this paper focuses the longitudinal validity of the BCTQ, which has not been investigated before in other languages.

## Materials and Methods

### Patients

Patients diagnosed with CTS at our outpatient clinic were consecutively included in our study. Diagnosis was based on clinical criteria as described previously (5). Patients underwent a neurological examination [including grip strength measurement with Martin Vigorimeter (21)], an electrodiagnostic examination (EDX), and ultrasonography (US) in a standardized way. The electrodiagnostic protocol has previously been described in more detail (22). Exclusion criteria were age under 18, a significant language barrier, a history or clinical signs of polyneuropathy or known hereditary neuropathy with liability to pressure palsies, previous trauma, or surgery to the wrist, a history of rheumatoid arthritis, diabetes mellitus, thyroid disease, alcoholism, arthrosis of the wrist, pregnancy, or severe atrophy of the abductor pollicis brevis muscle (APB). They all completed the Dutch version of the BCTQ at inclusion (T_1_) and about 6–8 months after CTR (T_2_). Permission from the local Medical Ethics Committee (Arnhem-Nijmegen) was obtained. The study was performed in accordance with the ethical standards of the 1964 Declaration of Helsinki. All subjects gave written informed consent in accordance with the Declaration of Helsinki.

### Boston Carpal Tunnel Questionnaire

The BCTQ was translated into Dutch. The Dutch translation is added in Supplementary Material. It consists of the Symptom Severity Scale (SSS) containing 11 questions, and it uses a five-point rating scale, and Functional Status Scale (FSS), which has 8 questions assessing the degree of complaints on a five-point scale. Mean sum scores of both scales were calculated and used for analysis. Measurement of symptoms or functional complaints is by definition measurement of a latent construct. For example, pain intensity in others cannot be observed but is rather inferred by taking a number of measurements on manifest characteristics (in the questionnaire), in this case on questions that try to capture how these complaints manifest (for example, how often are you awake during night due to pain in your wrist or hand?).

### Six-Point Likert Scale for Perceived Improvement

In addition to the BCTQ, at T_1_ and T_2_, patients rated their perceived treatment effect on a six-point Likert scale for perceived improvement, with the following grades: 1, “I am completely asymptomatic;” 2, “I very rarely have complaints;” 3, “I occasionally have complaints;” 4, “I often have complaints;” 5, “My complaints are the same as before treatment;” and 6, “My complaints have increased.”

### Analysis

A latent construct is a variable that is not directly observed, but is rather inferred from other variables that are directly measured by items on a questionnaire. Using multiple items reduces measurement error of the latent construct. Patients' position on the latent continuum is then quantified on a single scale by constructing a Likert scale, which is a weighted arithmetic average of the scores on all items. To do this, several conditions must be met. Firstly, the quality of Likert scales is determined by the extent to which they measure the severity of experienced symptoms in a *reliable* way. As is common practice, we assess scale reliability using Cronbach's α, which is a measure between 0 ≤ α ≤ 1 that quantifies the internal consistency of scales as a function of the number of items to be scaled, the average covariance between all item pairs, and the total variance. As a general rule of thumb, scales with α > 0.6 are considered adequate, and scales with α > 0.8 are considered reliable.

Secondly, Likert scales must be *unidimensional*, meaning that they consist of items that measure patients' scores on one single latent construct (construct validity). To assess whether the items on the SSS combine into a valid measure of the inherently unobservable severity of CTS symptoms, we used exploratory principal factor analyses (PFA). This approach uses patterns in the covariance matrix of a set of observable items to assess the number of underlying latent dimensions (factors). The first factor is drawn in such a way that it extracts maximum variance from the set of items, the second factor is extracted to tap the maximum of the remaining variance, and so forth. The variance explained by the factors is expressed as an eigenvalue. To determine the number of factors, we use an empirical criterion proposed by Kaiser (1960): we discard factors with an eigenvalue <1, which in practice means that we only interpret factors that explain more variance than single items do (23).

*Validity* was further analyzed with Spearman's correlations between the differences in SSS and the six-point Likert scale. The same was done for FSS. Moreover, BCTQ was correlated to electrophysiological severity according to Padua et al. (24), as well as grip strength. As is common practice, we also calculate changes in SSS and FSS between the baseline scores and follow-up (ΔSSS and ΔFSS, respectively) by subtracting the SSS and FSS score at follow-up from the SSS and FSS score at baseline (ΔSSS = SSS score at baseline—SSS score at follow-up). In all statistical analyses, a value of correlation coefficient between 0 and 0.25 was regarded as “no or weak” correlation, 0.26–0.50 was regarded as “moderate” correlation, 0.51–0.75 was regarded as “good” correlation, and 0.76–1.00 was regarded as “very good” correlation.

Thirdly, we need to gauge *responsiveness* of the scales. In order to do so, we evaluated the longitudinal validity of the measurement by testing for dimensionality pre- and post-treatment. Responsiveness was further assessed by calculating the effect size (ES = ΔSSS and FSS/SD of baseline score) and standard response mean (SRM = absolute ΔSSS and FSS/SD of change). An outcome measure should have a high SRM and high ES. According to Cohen, scores of ES and SRM are defined as small from ≥0.2 to <0.5, as medium from ≥0.5 to <0.8, and as large if ≥0.8 (25).

Finally, *acceptability* was assessed by calculating the response rate, and then ceiling and floor effect was assessed. The latter was investigated by calculating the percentages of respondents who achieved the lowest or highest possible scores on FSS and SSS at baseline. If more than 15% of participants achieved the lowest or highest possible scores, floor and ceiling effects were considered to be present. Comparison between baseline characteristics were analyzed by application of the chi-square test in case of categorical variables and the Mann–Whitney *U*-test for continuous variables with non-nominal distribution.

Statistical analyses were performed using SPSS Statistics 22.0.

## Results

A total of 229 patients were included in our study and underwent CTR. The SSS was completed by 192 patients at follow-up; the FSS, by 188 patients; and the six-point Likert scale for perceived improvement, by 195 patients. A total of 180 patients (143 women, 37 men) completed all questionnaires, and results of these patients were used. Median age was 50 (range 23–86). A total of 49 patients did not respond to all the questionnaires and where excluded. Baseline characteristics of the operated patients who did and did not complete the questionnaires are shown in Table 1. Pre- and post-scores on FSS and SSS of the included patients are shown in Table 2.

**Table 1:** Characteristics of the operated patients who completed all the questionnaires and where included in this study (*n* = 180) and those who did not completed the questionnaires and where excluded (*n* = 49).

	**Questionnaires completed**	Questionnaires not completed^*^	***p***
**Patient characteristics**			
Gender (female)	144 (80.0%)	34 (69.4%)	0.113
Age (median, range)	50 (23–86)	47 (18–84)	0.192
BMI (median, range)	26.3 (18.4–39.6)	26.8 (20.3–48.4)	0.924
Duration of symptoms in months (median, range)	18 (1–420)	12 (1–180)	0.086
**Electrodiagnostic test results (abnormal)**			
EDX	154 (85.6%)	38 (77.6%)	0.177
DML	124 (69.3%)	30 (62.5%)	0.372
DIG 1	148 (83.1%)	34 (70.8%)	0.056
DIG 4	155 (86.1%)	37 (77.1%)	0.127
PALM 2 or PALM 3	161 (89.4%)	41 (83.7%)	0.267
US	110 (62.1%)	23 (47.9%)	0.075
**Neurological examination**			
Atrophy APB muscle	24 (13.3%)	2 (4.2%)	0.076
Weakness APB muscle	49 (27.5%)	13 (27.7%)	0.986
Abnormal 2-point discrimination	120 (66.7%)	31 (66.0%)	0.927
Abnormal monofilament	72 (40.2%)	22 (46.8%)	0.415

* *Number of patients varies due to missing values*.

**Table 2:** Pre- and post-treatment scores of FSS and SSS and sensitivity to clinical change.

**Outcome**	**Pre-treatment**	**Post-treatment**	**Difference (Δ)**	**SRM**	**ES**
FSS ± SD (range)	2.30 ± 0.74 (1.00–5.00)	1.62 ± 0.72 (1.00–3.80)	0.68 ± 0.89	0.76	0.92
SSS ± SD (range)	2.95 ± 0.68 (1.30–4.80)	1.61 ± 0.74 (1.00–4.20)	1.34 ± 0.90	1.49	1.96

*FSS, Functional Status Scale; SSS, Symptom Severity Scale; Δ, difference between pre-treatment and post-treatment score; SRM, standard response mean; ES, effect size*.

### Reliability and Construct Validity

#### Functional Status Scale

Patients' functional capacity to perform certain tasks is measured with a battery of items from the FSS questionnaire. The items reflect the extent to which patients can perform different tasks (Table 3). Respondents were asked whether during a typical day, they had experienced problems when doing these tasks, as can be seen in the questionnaire in Supplementary Material. Patients could answer on a five-point scale: no problems, mild problems, rather serious problems, serious problems, and I cannot do it because of pain in hand or wrist.

**Table 3:** Explanation of items on FSS and SSS.

**Functional status scale**		**Symptom severity scale**	
**Item code**	**Scale item**	**Item code**	**Scale item**
FSS-1	Writing	SSS-1	Severity of nocturnal pain
FSS-2	Buttoning clothes	SSS-2	Frequency of nocturnal awakening due to pain
FSS-3	Holding a book	SSS-3	Severity of daytime pain
FSS-4	Holding a telephone	SSS-4	Frequency of daytime pain
FSS-5	Opening jars	SSS-5	Duration of daytime pain
FSS-6	Performing household chores	SSS-6	Severity of numbness
FSS-7	Carrying grocery bags	SSS-7	Severity of weakness
FSS-8	Bathing and getting dressed	SSS-8	Severity of tingling
		SSS-9	Severity of nocturnal numbness or tingling
		SSS-10	Frequency of nocturnal awakening due to numbness or tingling
		SSS-11	Difficulty with grasping and use of small objects

In Table 4, the results of the PFA are shown. In the first column, the communalities of the PFA are presented. These are the proportion of variance of the factors explained by each item. As a rule of thumb, items with low communalities (*h*^2^ ≤ 0.20) are considered to contribute too little to the factor solution. This is not the case (with exception of FSS-1).

**Table 4:** Reliability and dimensionality of scales based on items from the FSS battery.

	**A**			
	***h*^**2**^**	**Factor**	***h*^**2**^**	**Factor**
		**1**		**1**
**Items**				
FSS-1	0.161	0.401	0.383	0.619
FSS-2	0.317	0.563	0.566	0.753
FSS-3	0.408	0.639	0.584	0.764
FSS-4	0.304	0.552	0.454	0.674
FSS-5	0.361	0.601	0.619	0.787
FSS-6	0.610	0.781	0.739	0.860
FSS-7	0.558	0.747	0.710	0.842
FSS-8	0.471	0.687	0.577	0.759
**Eigenvalues**		3.750		5.034
**Model properties**				
Rotation^*^		None		None
Kaiser–Meier–Olkin		0.894		0.907
**Scale reliability**				
Cronbach's alpha	0.825			0.911

*N1 = 180, N2 = 180*.

* *Ob., Oblique rotation*.

The second column shows the factor loadings. These factor loadings can be interpreted as the correlation between the items and the various factors. The factor solution shows that all items are positively correlated with one factor. Generally, items that do not load strongly on a factor (with, say, a factor loading of *r* < 0.30) are not considered to load strongly enough to be included in the solution. This is also not the case in our study. The extracted factor has an eigenvalue of 3.750, which means it explains (3.750/11 × 100) = 34% of the variance on all 11 items. There are no other factors that have an eigenvalue >1. This suggests that the items refer to a single latent factor, which we label “Functionality.” A Likert scale based on these items shows a high internal consistency (Cronbach's α = 0.825). We constructed a Likert scale of these items, by computing the arithmetic mean over these items, while allowing missing values on two items.

In the final two columns, a similar PFA for the measurements post treatment is performed. The factor scores and communalities suggest that the scale is equally dimensional pre- and post-treatment, and that it is composed of the same underlying items.

#### Symptom Severity Scale

The severity of symptoms is also an unobservable construct. In order to assess how patients fare on this latent variable, we rely on the SSS questionnaire, designed to capture various aspects related to symptoms of CTS, and often used to measure their severity. The SSS battery as we used it, consisted of 11 items, measuring symptoms patients experience (Table 3). Respondents were asked to grade on a five-point scale the extent to which they experienced these symptoms, how often they experience them, or how severely they experienced these symptoms.

In Table 5A, we describe how the SSS items can be scaled, based on pre-treatment data. Table 5B concerns post-treatment data. In Model A of Table 5A, we test the initial solution with all items. Here, all items are allowed to correlate with all latent factors, and latent factors are assumed not to be correlated (no rotation). The model shows that a scale with all items has a high internal consistency (α = 0.847). However, the model also shows that the assumption of unidimensionality does not hold. Items appear to be associated with two factors that explain more variance than the separate items (eigenvalue > 1). All items explain a sufficiently high proportion of variance on the factors (*h*^2^ > 0.2). However, the solutions do not point toward a clear pattern: some of the items load on multiple factors. For example, SSS-9 is correlated with Factor 1 (*r* = 0.627) and with Factor 2 (*r* = 0.519).

**Table 5A:** Reliability and dimensionality of scales based on items from the SSS battery (before treatment).

	**A**				**B**			**C**		**D**		**E**			**F**		**G**	
	***h*^**2**^**	**Factor**			***h*^**2**^**	**Factor**		***h*^**2**^**	**Factor**	***h*^**2**^**	**Factor**	***h*^**2**^**	**Factor**		***h*^**2**^**	**Factor**	***h*^**2**^**	**Factor**
		**1**	**2**	**3**		**1**	**2**		**1**		**1**		**1**	**2**		**1**		**1**
**Items**																		
SSS-1	0.487	0.675	0.037	−0.174	0.462	0.652	0.193	0.233	0.483			0.477	0.668	−0.174	0.494	0.703		
SSS-2	0.712	0.726	−0.006	−0.429	0.816	0.804	0.413					0.755	0.733	−0.467	0.717	0.853		
SSS-9	0.682	0.627	0.519	−0.139								0.517	0.718	−0.042	0.427	0.654		
SSS-10	0.644	0.688	0.189	−0.368	0.593	0.671	0.377					0.624	0.719	−0.327	0.608	0.780		
SSS-3	0.585	0.638	−0.387	0.166	0.545	0.656	−0.339	0.547	0.740	0.547	0.740							
SSS-4	0.668	0.654	−0.490	−0.025	0.711	0.759	−0.369	0.722	0.850	0.712	0.844							
SSS-5	0.607	0.582	−0.518	0.001	0.627	0.678	−0.408	0.611	0.782	0.620	0.788							
SSS-8	0.433	0.513	0.394	0.120	0.159	0.348	0.195					0.374	0.582	0.188			0.271	0.520
SSS-11	0.530	0.569	0.058	0.451								0.474	0.547	0.417			0.528	0.726
SSS-6	0.346	0.401	0.289	0.320								0.334	0.454	0.357			0.317	0.563
SSS-7	0.488	0.548	0.075	0.426								0.464	0.542	0.412			0.502	0.708
**Eigenvalues**		4.487	1.633	1.341		3.491	1.215		2.534		2.284		3.628	1.336		2.675		
**Model properties**																		
Rotation^*^	None				Ob.			Ob.		Ob.		Ob.			Ob.			Ob.
Kaiser–Meier–Olkin	0.770				0.742			0.778		0.718		0.712			0.581			0.700
**Scale reliability**																		
Cronbach's alpha	0.847				0.822			0.788		0.805		0.826			0.835			0.723

* *Ob., Oblique rotation*.

**Table 5B:** Reliability and dimensionality of scales based on items from the SSS battery (after treatment).

	**H**			**I**		**J**	
	***h*^**2**^**	**Factor**		***h*^**2**^**	**Factor**	***h*^**2**^**	**Factor**
		**1**	**2**		**1**		**1**
**Items**							
SSS-1	0.669	0.816	0.063	0.614	0.783		
SSS-2	0.663	0.808	−0.102	0.671	0.819		
SSS-9	0.794	0.840	−0.296	0.773	0.879		
SSS-10	0.806	0.853	−0.279	0.787	0.887		
SSS-3	0.726	0.799	0.297			0.683	0.827
SSS-4	0.834	0.827	0.387			0.914	0.956
SSS-5	0.802	0.719	0.533			0.749	0.865
SSS-8	0.664	0.773	−0.256	0.637	0.798		
SSS-11	0.399	0.631	−0.026	0.395	0.629		
SSS-6	0.663	0.756	−0.302	0.640	0.800		
SSS-7	0.374	0.611	0.032	0.356	0.597		
**Eigenvalues**		6.839	1.096		5.232		2.556
**Model properties**							
Rotation^*^		None			Ob.		
Kaiser–Meier–Olkin		0.932			0.880		0.733
**Scale reliability**							
Cronbach's alpha		0.932			0.923		0.896

* *Ob., Oblique rotation*.

In Model B, we present the second solution, omitting the items that load on multiple factors. We also applied oblique rotation, to allow for the possibility that factors may be correlated. In this solution, that again has a high reliability (α = 0.822), two factors can be discerned. However, here too, some items (SSS-2, SSS-10, and SSS-8) load on both factors. Excluding these items from the solution (Model C) results in a unidimensional scale, with a reasonable reliability (α = 0.788). Additional empirical scrutiny shows that there are reasons to further purify the scale: omitting SSS-1 further increases the internal consistency (Model D: α = 0.805). The first factor therefore consists of the following items: SSS-3, SSS-4, and SSS-5. We assess that these items refer to *daytime symptoms*. In Model E, we repeat the routine with the remaining items. This model now shows a clearer pattern. The solution extracts two dimensions. SSS-1, SSS-2, SSS-9, and SSS-10 are positively correlated with the first factor and negatively correlated with the second factor. The other items clearly load on both factors. Model F presents the solution without these double loaders. The solution including just SSS-1, SSS-2, SSS-9, and SSS-10 produces a unidimensional scale (Model F), with a high internal consistency (α = 0.835) and a clear theoretical interpretability: it refers to *nighttime symptoms*. Finally, in Model G, we present a solution with the remaining items, SSS-8, SSS-11, SSS-6, and SSS-7. PFA extracts a single factor. The items are sufficiently highly internally consistent (α = 0.723), and they all refer to symptoms that relate to *operational capacity* of the hand.

Table 5B shows the result of the PFA post treatment. Interestingly, we arrive at a different factor solution. Model H demonstrates that the initial solution with all the items is composed of two orthogonal dimensions. A rotated solution without double loaders SSS-3, SSS-4, and SSS-5 has a unidimensional scale (Eigenvalue of 5.232) that includes the other items. In other words, the empirical evidence suggests that while pre-treatment, a clear empirical distinction between items referring to “nighttime symptoms” and those referring to the “operational capacity” of the hand could be discerned, post-treatment, SSS-1, SSS-2, SSS-9, SSS-10, SSS-8, SSS-11, SSS-6, and SSS-7 all refer to the same latent construct. While the items can still be separated based on theoretical considerations, the empirical evidence suggests that the resulting scales have a low longitudinal validity: they appear to measure different things pre- and post-treatment. This is not the case for a scale constructed for “daytime symptoms.” This scale has a high internal consistency, both pre- and post-measurement, is composed of the same items, and is unidimensional. It appears to have a sufficient longitudinal validity.

### Validity

The ΔFSS and ΔSSS had good correlation with the six-point Likert scale for perceived improvement (*r* = 0.524; *p* < 0.01 and 0.574; *p* < 0.01, respectively), indicating that the more improvement patients perceived, the more improvement was seen on FSS and SSS (Table 6). Moreover, there was a moderate correlation between FSS and pinch grip (*r* = 0.259; *p* < 0.01) and a weak correlation between SSS and pinch grip (*r* = 0.231; *p* < 0.01). No correlation could be demonstrated with electrophysiological severity or ultrasonography.

**Table 6:** Correlation between BCTQ and other outcome measures.

**Scale**	**FSS**		**SSS**	
	***r***	***p***	***r***	***p***
Six-point Likert scale	0.524^#^	<0.01	0.574^†^	<0.01
Grip strength	0.259	<0.01	0.231	<0.01
Electrophysiological severity	0.014	0.857	−0.006	0.932
Ultrasonography	0.007	0.925	0.087	0.249

# *ΔFSS is used*.

† *ΔSSS is used*.

*r, Spearman's rank correlation coefficient*.

### Responsiveness

For FSS, an SRM of 0.76 and an ES of 0.92 was found. For SSS, SRM was 1.49 and ES was 1.96 (Table 2).

### Acceptability and Ceiling and Floor Effect

Response rate for FSS was 82.1%, and for SSS, it was 83.8%. In FSS, a total of five patients (2.3%) had a score of 1, and two (1.1%) had a score of 5 pre-treatment. In SSS, no patients had a score of 1 or 5 (Table 2).

## Discussion

Several reports have shown that the BCTQ in many different languages has excellent psychometric properties. In this study, we have demonstrated that the Dutch version of the BCTQ also has a proper reliability, validity, responsiveness, and acceptability. However, longitudinal (the use after an intervention) validity for the SSS subscale seems to be insufficient. Also, the assumption of unidimensionality is violated.

The Dutch version of the BCTQ has shown a good reliability. Internal consistency is high with Cronbach α = 0.825 for FFS and 0.847 for SSS, which is comparable to the Spanish, Portuguese, Swedish, Turkish, Japanese Greek, and original versions (6, 12–16, 26).

Factor analysis showed that FSS is a unidimensional scale. In other words, all items in the FSS refer to a single latent factor, which relates to “functionality.” However, the SSS measures three different concepts, namely, “daytime symptoms,” “nighttime symptoms,” and “operational capacity.” Internal consistency of these three different subsets of items is, however, also high, with Cronbach α = 0.805, 0.835, and 0.723, respectively. It is important to realize that the SSS sum score consists of these different concepts. The assumption of unidimensionality does not hold in the SSS, which is similar to three previously reported studies that have subjected the SSS to factor analysis. The Japanese version extracted “daytime pain” as the first factor (consisting items SSS-3, 4, and 5) and the other factors as the second factor. They stated that, on a theoretical basis, item SSS-11 (difficulty with grasping) could be included in the FSS (26). Artroshi et al. performed a factor analysis on the Swedish version, combining FSS and SSS. They found the BCTQ consists of three factors, namely, “functionality” (consisting FSS-1–8 and SSS-7 and SSS-11), “nighttime symptoms and numbness/tingling” (consisting SSS-1, 2, 6, 8, 9, and 10), and “daytime symptoms” (consisting SSS-3, 4, and 5) (27). In the Portuguese version, five different factors could be identified after combining FSS and SSS (“weakness,” consisting of SSS-7, FSS-5, 6, 7, and 8; “paresthesias,” consisting of SSS-6, 8, 9, and 10; “pain,” consisting of SSS-3, 4, and 5; “nocturnal symptoms,” consisting of SSS-1, 2, and 10; “disability,” consisting of SSS-11, FSS-2, 3, and 4) (13).

Validity is the extent to which the questionnaire appears to measure what it purports to measure. Longitudinal validity of the FSS is adequate. The scale is unidimensional pre- as well as post-treatment and can therefore be used for interpretation of pre- and post-treatment differences in “functionality.” However, longitudinal validity of the SSS turned out to be insufficient, as it consists of three dimensions pre-treatment and two dimensions post-treatment. It is somewhat difficult to understand why “daytime symptoms” and “operational capacity” form one construct post-treatment. A possible reason for change in the factor structure post-treatment could be the fact that the patients post-treatment are “different” compared to the patients pre-treatment. They have undergone surgery and therefore they can have other complaints that cannot be strictly attributed to CTS (e.g., wound pain, swelling, wrist instability), which can be of influence when completing the questionnaire. Moreover, these findings are based on a sample of only 180 patients. These problems may be eliminated in future research using a greater sample size and by only selecting patients who have persisting CTS complaints postoperatively. In addition, another solution to possibly overcome this problem is to test and retest the FSS and SSS in a sample of CTS patients not undergoing CTR (or on a waiting list for CTR). We performed an analysis in 34 patients initially not operated and treated conservatively. However, the number of patients seems to be too low to draw firm conclusions from this. There is no previous research on longitudinal validity of the BCTQ. Therefore, caution is advised when applying the SSS postoperatively. Construct validity is often assessed quantitatively by examining the correlation coefficients between the instrument in question and other measures (28). Validity of the Dutch version of the BCTQ was demonstrated by correlating the differences in FSS and SSS with a six-point Likert scale for perceived improvement (*r* = 0.524; *p* < 0.01 and *r* = 0.574; *p* < 0.01). Similar results were found by Bessette et al. who found good correlation between the BCTQ and extent of symptom relief (*r* = 0.51) and patients' satisfaction (*r* = 0.56) (29). As demonstrated in previous studies, there is a correlation between BCTQ and grip strength, but no correlation between BCTQ and electrophysiological severity (6, 10, 13, 15). However, as dimensionality of SSS post-treatment differs from pre-treatment, conclusions based on these correlations should be drawn with care. Responsiveness refers to the ability of a measure to detect change accurately when it had occurred (30). It is assessed by measuring the magnitude of change in scores, which occurs over time and as a result of an intervention, e.g., surgical release (10, 31). Our results showed that the BCTQ has moderate to large ES and SRM. In other words, the BCTQ is sensitive to clinical change. In previous research, varying values are reported, broadly in line with our results (6–8, 16, 26, 32–34). Again, the interpretation of these values is questionable given the insufficient longitudinal validity. Responsiveness should not be confused with the interpretability of the scale. Interpretability is assessed by “minimal clinically important difference” (MCID). For the Dutch version of the BCTQ, we recently described the MCID (35).

The BCTQ had shown good acceptability. However, acceptability of our version was somewhat lower than in most previously reported literature, in which loss to follow-up or incomplete responses ranged from 1 to 10% (8, 16, 29, 32). Response rates comparable to ours have also been reported before (33, 34). Finally, no significant floor or ceiling effects were observed, as for the FSS and SSS, very low number of patients had lowest or highest scores (for FSS: lowest score 2.3% and highest score 1.1%; for SSS, no patients had highest or lowest score).

The main strengths of our study are the relatively big sample size, extensiveness of psychometric properties that have been analyzed, and the application of PFA to determine construct validity. As a limitation of the present study, it can be designated that the six-point Likert scale we used as a validation method of the BCTQ is a self-developed scale that has not been validated before. However, such a scale has been applied in previous studies (29). Moreover, we did not assess reproducibility. Thirdly, our Dutch translation of the BCTQ was not back translated to the English language to check for consistency for the original version.

In conclusion, we demonstrated that the Dutch version of the BCTQ has a proper reliability, validity, responsiveness, and acceptability to assess the symptom severity and functional disabilities of CTS patients. Besides, we showed that the FSS is unidimensional. However, in our sample, the SSS consists of multiple dimensions. Moreover, the meaning that patients attribute to items in the SSS battery changed pre- and post-surgery. This raises questions on the extent to which SSS can be used to compare symptom severity before and after a clinical intervention. Therefore, in future clinical trials that also find multiple dimensions in the SSS items, we would recommend to create sum scores of the different dimensions of the SSS, instead of constructing Likert scales of all items in the battery. Moreover, because of the insufficient longitudinal validity of the SSS, caution is required when interpreting the results postoperatively.

## Data Availability Statement

The datasets generated for this study are available on request to the corresponding author.

## Ethics Statement

The studies involving human participants were reviewed and approved by Medical Ethics Committee (Arnhem-Nijmegen). The patients/participants provided their written informed consent to participate in this study.

## Author Contributions

FD organized the database. ML and FD performed the statistical analysis and wrote the first draft of the manuscript. All authors contributed to the conception and design of the study, manuscript revision, and read and approved the submitted version.

### Conflict of Interest

The authors declare that the research was conducted in the absence of any commercial or financial relationships that could be construed as a potential conflict of interest.
