# Supplementary material for: Validation of the Dutch Version of the Boston Carpal Tunnel Questionnaire
Source: Front Neurol. 2019 Nov 7;10:1154. doi: 10.3389/fneur.2019.01154 (PMC6854027; doi:10.3389/fneur.2019.01154)
Supplement: Supplementary file 1 [file Table_1.DOCX]

Supplementary Material

# Supplementary Data

**VRAGENLIJST 1**

De volgende vragen hebben betrekking om de ernst van uw klachten gedurende een kenmerkende dag in de afgelopen 2 weken. Graag het antwoord dat op u van toepassing is omcirkelen:

**Klachtenscore (Symptom Severity Score (SSS))**

| **1** | **Hoe ernstig is de pijn in de hand of pols, die u ’s nachts hebt?** | |
| --- | --- | --- |
|  | **1** | Ik heb geen pijn in mijn hand of pols ’s nachts |
|  | **2** | De pijn is mild |
|  | **3** | De pijn is behoorlijk |
|  | **4** | De pijn is ernstig |
|  | **5** | De pijn is nauwelijks te verdragen |
|  | | |
| **2** | **Hoe vaak wordt u wakker van de pijn gedurende de nacht?** | |
|  | **1** | Nooit |
|  | **2** | Eenmaal per nacht |
|  | **3** | Twee- of driemaal per nacht |
|  | **4** | Vier- of vijfmaal per nacht |
|  | **5** | Vaker dan vijfmaal per nacht |
|  | | |
| **3** | **Heeft u overdag ook pijn in uw hand of pols?** | |
|  | **1** | Ik heb nooit pijn overdag |
|  | **2** | Ik heb milde pijn overdag |
|  | **3** | Ik heb behoorlijk veel pijn overdag |
|  | **4** | Ik heb ernstige pijn overdag |
|  | **5** | Ik heb nauwelijks te verdragen pijn overdag |
|  | | |
| **4** | **Hoe vaak heeft u pijn overdag?** | |
|  | **1** | Nooit |
|  | **2** | Eenmaal per dag |
|  | **3** | Twee- of driemaal per dag |
|  | **4** | Vier- of vijfmaal per dag |
|  | **5** | Vaker dan vijfmaal per dag |
|  | | |
| **5** | **Hoe lang duurt zo’n periode met pijn gemiddeld overdag?** | |
|  | **1** | Ik heb geen pijn overdag |
|  | **2** | Minder dan 10 minuten |
|  | **3** | 10 minuten tot een uur |
|  | **4** | Meer dan een uur |
|  | **5** | De pijn is constant aanwezig overdag |
|  | | |
| **6** | **Heeft u een doof gevoel of gevoelloosheid in uw hand?** | |
|  | **1** | Nee |
|  | **2** | Ik heb een beetje een doof gevoel in mijn hand |
|  | **3** | Ik heb een behoorlijk doof gevoel in mijn hand |
|  | **4** | Ik heb een ernstig doof gevoel |
|  | **5** | Ik heb een zeer ernstig doof gevoel |

| **7** | **Heeft u krachtsverlies (zwakte) van de hand?** | |
| --- | --- | --- |
|  | **1** | Nee |
|  | **2** | Ja, iets zwakte |
|  | **3** | Ja, behoorlijke zwakte |
|  | **4** | Ja, ernstige zwakte |
|  | **5** | Ik kan bijna niets meer met de hand |
|  | | |
| **8** | **Heeft u tintelingen of een slapend gevoel in uw hand?** | |
|  | **1** | Nee |
|  | **2** | Ja, milde tintelingen |
|  | **3** | Ja, behoorlijk wat tintelingen |
|  | **4** | Ja, ernstig veel tintelingen |
|  | **5** | Ja, zeer ernstige tintelingen |
|  | | |
| **9** | **Hoe ernstig zijn het dove gevoel of de tintelingen ’s nachts?** | |
|  | **1** | Ik heb ’s nachts geen doof gevoel of tintelingen |
|  | **2** | De klacht is mild |
|  | **3** | De klacht is behoorlijk |
|  | **4** | De klacht is ernstig |
|  | **5** | De klacht is zeer ernstig |
|  | | |
| **10** | **Hoe vaak wordt u wakker van een doof gevoel of tintelingen?** | |
|  | **1** | Nooit |
|  | **2** | Eenmaal |
|  | **3** | Twee- of driemaal |
|  | **4** | Vier- of vijfmaal |
|  | **5** | Meer dan vijfmaal |
|  | | |
| **11** | **Hebt u problemen met het vastpakken van kleine voorwerpen, bv sleutel of balpen?** | |
|  | **1** | Geen |
|  | **2** | Een beetje problemen |
|  | **3** | Behoorlijk wat problemen |
|  | **4** | Ernstige problemen |
|  | **5** | Ik kan zulke voorwerpen helemaal niet meer vastpakken |

**Vragenlijst 2**

De volgende vragen hebben betrekking op een kenmerkende dag gedurende de afgelopen twee weken. Hebt u gedurende zo een kenmerkende dag wel eens moeite gehad om een van de volgende bezigheden uit te voeren? Omcirkel a.u.b. het getal dat het beste omschrijft in welke mate u in staat was de activiteiten te verrichten.

Functionele score (Functional Status Score (FSS))

|  | | | | | |
| --- | --- | --- | --- | --- | --- |
| **Activiteit** | **Geen problemen** | **Een beetje problemen** | **Behoorlijke problemen** | **Ernstige problemen** | **Ik kan het helemaal niet meer door klachten hand/pols** |
| *Schrijven* | 1 | 2 | 3 | 4 | 5 |
| *Knoopjes vastmaken* | 1 | 2 | 3 | 4 | 5 |
| *Een boek vasthouden tijdens lezen* | 1 | 2 | 3 | 4 | 5 |
| *Een telefoon vasthouden* | 1 | 2 | 3 | 4 | 5 |
| *Potjes openmaken* | 1 | 2 | 3 | 4 | 5 |
| *Huishoudelijk werk verrichten* | 1 | 2 | 3 | 4 | 5 |
| *Boodschappentassen dragen* | 1 | 2 | 3 | 4 | 5 |
| *Baden/aankleden* | 1 | 2 | 3 | 4 | 5 |
